# Inter-rater reliability of seven neurolaryngologists in laryngeal EMG signal interpretation

**DOI:** 10.1007/s00405-019-05553-y

**Published:** 2019-07-16

**Authors:** Guan-Yuh Ho, Matthias Leonhard, Gerd Fabian Volk, Gerhard Foerster, Claus Pototschnig, Kathleen Klinge, Thordis Granitzka, Anne-Kristin Zienau, Berit Schneider-Stickler

**Affiliations:** 1 Division of Phoniatrics-Logopedics, Department of Otorhinolaryngology, Medical University of Vienna, Waehringer Guertel 18–20, 1090 Vienna, Austria; 2 Department of Otorhinolaryngology, Jena University Hospital, Jena, Germany; 3 Department of Otorhinolaryngology, SHR Wald-Klinikum Gera, Gera, Germany; 4 Department of Otorhinolaryngology, University of Innsbruck, Innsbruck, Austria

**Keywords:** Laryngeal electromyography, Inter-rater reliability, Vocal fold paresis, Laryngology, Kappa statistic

## Abstract

**Purpose:**

Laryngeal electromyography (LEMG) has been considered as gold standard in diagnostics of vocal fold movement impairment, but is still not commonly implemented in clinical routine. Since the signal interpretation of LEMG signals (LEMGs) is often a subjective and semi-quantitative matter, the goal of this study was to evaluate the inter-rater reliability of neurolaryngologists on LEMGs of volitional muscle activity.

**Methods:**

For this study, 52 representative LEMGs of 371 LEMG datasets were selected from a multicenter registry for a blinded evaluation by 7 experienced members of the neurolaryngology working group of the European Laryngological Society (ELS). For the measurement of the observer agreement between two raters, Cohen’s Kappa statistic was calculated. For the interpretation of agreements of diagnoses among the seven examiners, we used the Fleiss’ Kappa statistic.

**Result:**

When focusing on the categories “no activity”, “single fiber pattern”, and “strongly decreased recruitment pattern”, the inter-rater agreement varied from Cohen’s Kappa values between 0.48 and 0.84, indicating moderate to near-perfect agreement between the rater pairs. Calculating with Fleiss’ Kappa, *a* value of 0.61 showed good agreement among the seven raters. For the rating categories, the Fleiss’ Kappa value ranged from 0.52 to 0.74, which also showed a good agreement.

**Conclusion:**

A good inter-rater agreement between the participating neurolaryngologists was achieved in the interpretation of LEMGs. More instructional courses should be offered to broadly implement LEMG as a reliable diagnostic tool in evaluating vocal fold movement disorders in clinical routine and to develop future algorithms for therapy and computer-assisted examination.

## Introduction

In 1944, Weddell et al. [1] introduced the laryngeal electromyography (LEMG). In the 1950s, Faaborg-Anderson et al. researchers noticeably further advanced it [2–8]. During the 1980s and 1990s, diagnosis and treatment of voice disorders have been described using various tools based on laryngeal LEMG.

Until now, in most ENT or phoniatric departments LEMG is still not commonly used in clinical routine. Internationally, clinicians still mainly use laryngoscopy and stroboscopy for diagnosing vocal fold paralysis/paresis. According to Wu et al. [9], only 1.7% of the otolaryngologists responded to use LEMG for the diagnosis of vocal fold paresis (VFP) in the US. Similar result was also presented for Europe by Volk et al. [10], with only 3.6% of the responding that LEMG is the most important tool for diagnosing VFP. This may be due to the lack of agreement on methodology, interpretation, validity, and clinical application of LEMG when using LEMG [11–16]. To minimize these problems, guidelines for using LEMG have been developed, and complemented with workshops on LEMG. Also partnerships with neurological departments have been established to share knowledge and join efforts to promote the implementation into clinical routine. A working group on neurolaryngology of the European Laryngological Society (ELS) is dealing with the evaluation of existing guidelines for LEMG performance and for the identification of issues requiring further clarification [12]. The primary assignment of the working group was to teach the key techniques of LEMG surgery. The group published a proposal for a set of recommendations for LEMG and initiated a registry with the aim of collecting LEMG data recorded according to these published recommendations [12, 17]. Meetings and workshops have been organized for participants of the registry and other professionals interested in LEMG and neurolaryngology with the aim of providing a sufficient level of standardization and data quality.

Critical clinical information on the electrophysiologic status of the larynx can be reliably obtained by LEMG [18]. Beside the diagnosis of most neuromuscular diseases of the larynx, some clinicians consider LEMG as the required diagnostic tool for certain neuromuscular disorders of the larynx, such as VFP [18].

The initial diagnosis of respiratory immobility of the vocal fold is made during laryngeal examination, when there is a reduction or an absence of abduction or adduction of the true vocal fold as seen during laryngeal examination. Laryngeal paralysis is the most frequent cause of the vocal fold immobility. For its diagnosis, LEMG is an important diagnostic tool, particularly when performed 10–14 days after the onset of vocal fold immobility [13]. A diagnosis of arytenoid fixation is based on normal electrical activity patterns of the LEMG [19], while abnormal electrical activity patterns, including patterns of denervation or reinnervation, support the diagnosis of vocal fold paralysis [20].

Interpretation of LEMG signals comprises the recognition and evaluation of patterns, such as insertion activity, spontaneous activity, fibrillations, positive sharp waves, polyphasic action potentials and motor unit potential (MUP) recruitment. Appearance and interpretation of these signals also depend on the grade of volitional agonistic and antagonistic muscle activation during the evaluation. The absence of spontaneous activity, fibrillations or positive sharp waves, and presence of good motor recruitment, with or without polyphasic action potentials in LEMGs are signs for excellent prognosis.

To fully encompass the cause of vocal fold disorders using LEMG, signal recordings of the thyroarytenoid (TA), cricoarytenoid (CT) and posterior cricoarytenoid (PCA) muscles, which are innervated by the recurrent laryngeal nerve (RLN), are recommended for evaluation.

In case of RLN injury, the larynx is rarely totally denervated or paralyzed. Notably, adductor and abductor axons, as well as sensory and autonomic fibers run interwoven within the common trunk of the RLN [21]. When the laryngeal nerve is injured, the regeneration of these nerve components takes place to various degrees. Improper axonal redirection of nerve fibers into inappropriate muscle is possible and may occur in nerve trunks that supply multiple muscles [22]. This abnormal reinnervation is called synkinesis [23]. Actually, electromyography (EMG) typically reveals evidence of muscle activity despite the functional finding of immobility [24, 25]. Crumley has extensively discussed the imperfect regenerative ability of the RLN [21, 23, 26, 27].

The neurolaryngologist can draw conclusions on the functionality of the axons and neuromuscular junctions by interpretation of the LEMG signals that are acquired by needle electrodes placed in each target muscle tested. However, in the absence of reliable computer-assisted signal quantification methods, the interpretation of LEMGs remains based on subjective recognition of descriptive characteristics by each individual examiner. Thus, since the interpretation of LEMG seems partly a subjective matter and likely depends on training and experience level of the rater, the inter-rater agreement on diagnosing LEMGs is of particular interest and objective of the presented study.

In the first evaluation step, the examiners analyzed and classified the selected LEMGs. In the second step, the classification of the examiners was initially tested against each other using the Cohen’s Kappa. Then, the results of the examiners’ evaluation were analyzed using the Fleiss’ Kappa.

Historically, percent agreement (number of agreement on a rating/total number of ratings) was used to determine inter-rater reliability [28]. However, chance agreement due to raters guessing is possible. To take this element of chance into account, in 1960, Jacob Cohen proposed the kappa statistic to provide more accurate measurement of the reliability between two raters making decisions about how a particular unit of analysis should be categorized. Cohen’s Kappa measures the percentage of agreement between two raters and calculates the degree to which agreement can be accredited to chance [29]. For assessing the observer agreement between more than two raters, Joseph Fleiss proposed the generalization of unweighted kappa [30]. It is to mention that Fleiss’ Kappa, one of the most common indices to quantify multiple-raters agreement [31], is the extension of William Scott’s *π* index [32, 33].

## Methods

From May 2012 to March 2014, laryngologists from 14 different European clinical departments with special interest in neurolaryngology joined a multicenter registry to collect LEMG datasets, and to learn more about the indications for performing LEMG and the interpretation of the results. The local ethics committees gave approval in all participating hospitals (Ethical Committee of the University Department of Jena, No. 5145-04/17). The departments had the possibility to send staff experts to perform LEMG together [34].

For this study, seven experienced neurolaryngologists from Germany and Austria—five otolaryngologists and two phoniatricians—have been selected to evaluate pre-recorded LEMG data according to the guidelines of the European Laryngological Society [17]:

1. No activity/electric silence.
2. Single fiber activity/single fiber pattern.
3. Strongly decreased recruitment patterns.
4. Mildly decreased recruitment pattern.
5. Normal/dense recruitment pattern.

Only signal recordings of maximum volitional activity of single muscles, that have been acquired during agonistic maneuvers, were included in this study, while evaluation of possible synkinetic reinnervation of several muscles were not part of the study.

From a multicenter LEMG registry consisting of 371 LEMG datasets, 52 representative LEMGs have been selected as not all LEMGs in the registry were usable for a study purpose due to insufficient length and shape of the LEMG recordings. From the 52 selected LEMGs, 26 LEMGs referred to LEMGs of the thyroarytenoid muscle (TA), 21 to posterior cricoarytenoid muscle (PCA) and 5 to cricothyroid muscle (CT). The evaluation of the selected LEMGs was blinded, since the examiners had no knowledge about the original classification of the selected LEMGs.

In this evaluation study, Cohen’s Kappa was used to measure the agreement between two raters who each classify *N* LEMG samples into *C* equally exclusive categories. Cohen’s Kappa statistic measures inter-rater reliability. Inter-rater reliability happens when data raters give the same score to the same data item.

The kappa statistic varies from 0 to 1. The kappa results could be interpreted as shown in Table 1.

**Table 1:** Interpretation of Cohen’s Kappa value

Cohen’s Kappa value	Interpretation of Cohen’s Kappa value
≤ 0	No agreement
0.1–0.20	Slight agreement
0.21–0.40	Fair agreement
0.41–0.60	Moderate agreement
0.61–0.80	Substantial agreement
0.81–0.99	Near-perfect agreement
1	Perfect agreement

To calculate Cohen’s Kappa, following formula was used:

$$k=\frac{P_{\rm a}-P_{\rm e}}{1-P_{\rm e}}$$

*P*_a_ represents the actual observed proportion of agreement and *P*_e_ the proportion of agreement expected by chance. *P*_*a*_ is calculated by the LEMG diagnoses in agreement per total number of subjects (LEMG samples). Since the kappa is based on the Chi square table, the value of *P*_e_ can be calculated with the following formula [35]:

$$\mathrm{E}\mathrm{x}\mathrm{p}\mathrm{e}\mathrm{c}\mathrm{t}\mathrm{e}\mathrm{d}\mathrm{ }\left(\mathrm{c}\mathrm{h}\mathrm{a}\mathrm{n}\mathrm{c}\mathrm{e}\right)\mathrm{ }\mathrm{a}\mathrm{g}\mathrm{r}\mathrm{e}\mathrm{e}\mathrm{m}\mathrm{e}\mathrm{n}\mathrm{t}=\frac{\left(\frac{cm1 x rm1}{n}\right)+\dots +\left(\frac{cm4 x rm4}{n}\right)}{n}$$

Since Cohen’s Kappa is only suitable for evaluating the inter-rater reliability between two raters, we further used the Fleiss’ Kappa [30] to obtain the values for interpreting the agreements of diagnoses among the 7 examiners for their expertise opinions on the selected 52 LEMG samples.

In our study, we had ***N*** LEMG samples and *r* rates per subject. All raters had to assign each LEMG sample in one of the ***C*** mutually exclusive categories. The LEMG samples were represented by the subscript *i*, where *i* = 1, …, 52, and the categories of the scale by the subscript *j*, where *j* = 1, …, 4.

The number of the raters who assigned the *i*th LEMG sample to the *j*th category was defined as *r*_*ij*_. The proportion of all assignments to the *j*th category was defined as *p*_*j*_, which according to Scott [32] and Fleiss [30] is

$${p}_{j}=\frac{1}{Nr}\sum_{i=1}^{C}{r}_{ij}.$$

And the proportion of pair of raters agreeing in the *i*th subject was defined as* P*_***i***_, which is

$${P}_{i}=\frac{1}{r(r-1)}\sum_{j=1}^{C}{r}_{ij}({r}_{ij}-1)=\frac{1}{r\left(r-1\right)}\left(\sum_{j=1}^{C}{r}_{ij}^{2}-r\right).$$

The overall extent of agreement measured by the mean of the *P*_*i*_s as proposed by Fleiss [30] and Fleiss et al. [31] is, therefore,

$$\bar{P}=\frac{1}{N}\sum_{i=1}^{N}{P}_{i}=\frac{1}{Nr(r-1)}\left(\sum_{i=1}^{N}\sum_{j=1}^{C}{r}_{ij}^{2}-Nr\right)$$

The mean proportion of agreement, as proposed by Scott [32] and Fleiss [30],meaning for both categories from

$${\bar{P}}_{e}=\sum_{J=1}^{C}{p}_{j}^{2},$$

measures the degree of agreement based on chance.

As suggested by Fleiss [30], we obtained the kappa statistic by correcting the overall extent of agreement for the mean proportion of agreement based on chance and normalized:

$${k}_{\mathrm{F}\mathrm{l}\mathrm{e}\mathrm{i}\mathrm{s}\mathrm{s}}=\frac{\bar{P}-{\bar{P}}_{e}}{1-{\bar{P}}_{e}}$$

To measure the extent of agreement beyond chance in assignment to category *j* proposed by Fleiss [30], the following formula was used:

$${k}_{j}=\frac{\sum_{1=1}^{N}{r}_{ij}^{2}-Nr{p}_{j}\left[1+\left(r-1\right){p}_{j}\right]}{Nr\left(r-1\right){p}_{j}\left(1-{p}_{j}\right)}$$

For the interpretation of kappa coefficient, Fleiss proposed the categories: poor agreement (*k*_Fleiss_ < 0.40), good agreement (*k*_Fleiss_ between 0.40–0.75) and excellent agreement (*k*_Fleiss_ > 0.75) [30].

## Results

To interpret the inter-rater reliability between two raters, we have performed the calculation of Cohen’s Kappa. The kappa results from the seven examiners against each other can be found in Table 2.

**Table 2:** Cohen’s Kappa values between rater pairs

Cohen’s Kappa value							
vs.	Rater 1	Rater 2	Rater 3	Rater 4	Rater 5	Rater 6	Rater 7
Rater 1		0.73	0.48	0.84	0.50	0.53	0.65
Rater 2	0.73		0.54	0.74	0.51	0.61	0.61
Rater 3	0.48	0.54		0.53	0.69	0.61	0.63
Rater 4	0.84	0.74	0.53		0.48	0.64	0.76
Rater 5	0.50	0.51	0.69	0.48		0.57	0.60
Rater 6	0.53	0.61	0.61	0.64	0.57		0.66
Rater 7	0.65	0.61	0.63	0.76	0.60	0.66	

As shown in Table 2, when comparing the results of the raters against each other, the Cohen’s Kappa value ranges from 0.48 to 0.84, which means from moderate agreement to near-perfect agreement. Calculation of the rater pairs using Cohen’s Kappa, 42.86% achieved a moderate agreement and 52.38% a substantial agreement, whereas 4.76% of the rater pairs reached near-perfect agreement.

The inter-rater agreement of rater pairs for each category is presented in Table 3. “Category reliability between rater pairs”.

**Table 3:** Category reliability between rater pairs

Cohen’s Kappa value							
vs.	Rater 1	Rater 2	Rater 3	Rater 4	Rater 5	Rater 6	Rater 7
*1. No activity/electric silence*							
Rater 1		0.96	0.66	0.82	0.61	0.67	0.71
Rater 2	0.96		0.61	0.86	0.55	0.62	0.66
Rater 3	0.66	0.61		0.71	0.95	0.83	0.87
Rater 4	0.82	0.86	0.71		0.71	0.77	0.81
Rater 5	0.61	0.55	0.95	0.71		0.87	0.91
Rater 6	0.67	0.62	0.83	0.77	0.87		0.86
Rater 7	0.71	0.66	0.87	0.81	0.91	0.86	

Cohen’s Kappa value							
vs.	Rater 1	Rater 2	Rater 3	Rater 4	Rater 5	Rater 6	Rater 7
*2. Single fiber activity*							
Rater 1		0.67	0.44	0.87	0.36	0.39	0.60
Rater 2	0.67		0.52	0.68	0.44	0.50	0.52
Rater 3	0.44	0.52		0.39	0.83	0.55	0.63
Rater 4	0.87	0.68	0.39		0.30	0.37	0.63
Rater 5	0.36	0.44	0.83	0.30		0.55	0.55
Rater 6	0.39	0.50	0.55	0.37	0.55		0.49
Rater 7	0.60	0.52	0.63	0.63	0.55	0.49	

Cohen’s Kappa value							
vs.	Rater 1	Rater 2	Rater 3	Rater 4	Rater 5	Rater 6	Rater 7
*3. Strongly decreased recruitment pattern*							
Rater 1		0.57	0.39	0.89	0.50	0.52	0.61
Rater 2	0.57		0.57	0.65	0.56	0.69	0.60
Rater 3	0.39	0.57		0.46	0.50	0.44	0.39
Rater 4	0.89	0.65	0.46		0.39	0.62	0.70
Rater 5	0.50	0.56	0.50	0.39		0.44	0.43
Rater 6	0.52	0.69	0.44	0.62	0.44		0.56
Rater 7	0.61	0.60	0.39	0.70	0.43	0.56	

Cohen’s Kappa value							
vs.	Rater 1	Rater 2	Rater 3	Rater 4	Rater 5	Rater 6	Rater 7
*4. Mildly decreased recruitment pattern*							
Rater 1		0.70	0.42	0.74	0.63	0.63	0.74
Rater 2	0.70		0.52	0.84	0.56	0.74	0.84
Rater 3	0.42	0.52		0.68	0.47	0.73	0.68
Rater 4	0.74	0.84	0.68		0.61	0.92	1.00
Rater 5	0.63	0.56	0.47	0.61		0.51	0.61
Rater 6	0.63	0.74	0.73	0.92	0.51		0.92
Rater 7	0.74	0.84	0.68	1.00	0.61	0.92	

For the category “no activity/electric silence”, the kappa value ranges from 0.55 to 0.96, meaning from moderate agreement to near-perfect agreement. The kappa values for the categories “single fiber activity” and “strongly decreased recruitment pattern” range from 0.30 to 0.87 and from 0.39 to 0.89, respectively, meaning for both categories from fair agreement to near-perfect agreement. As for the category “mildly decreased recruitment pattern”, the kappa value lies between 0.42 and 1, which means between moderate agreement and perfect agreement.

In other words, assessing the inter-rater agreement of rater pairs regarding the given categories using Cohen’s Kappa, in the category “no activity/electric silence” 14.29% of rater pairs achieved moderate agreement, 38.09% substantial agreement and 47.62% near-perfect agreement. In the category “single fiber activity”, fair agreement was achieved by 23.81%, moderate agreement by 47.62%, substantial agreement by 19.05% and near-perfect agreement by 9.52% of rater pairs. For the category “strongly decreased recruitment pattern”, 14.29% of rater pairs achieved fair agreement, 57.14% moderate agreement, 23.81% substantial agreement and 4.76% near-perfect agreement. And in the category “mildly decreased recruitment pattern”, 23.81% of rater pairs achieved moderate agreement, 52.38% substantial agreement, 19.05% near-perfect agreement and 4.76% perfect agreement.

As seen in Table 4, we had 52 LEMG samples in our study. For each LEMG sample, we had seven ratings. Thus, for the evaluation of overall agreement among the seven examiners, we used the formula described by Fleiss to calculate the kappa value and got a result of 0.61. It means there was a good agreement among the seven raters in their expert opinion on the selected LEMGs.

**Table 4:** Diagnoses of 52 LEMG samples by 7 raters

LEMG samples (*i*)	Category				LEMG samples (*i*)	Category			
	No activity/electric silence (*j* = 1)	Single fiber activity (*j* = 2)	Strongly decreased recruitment pattern (*j* = 3)	Mildly decreased recruitment pattern (*j* = 4)		No activity/electric silence (*j* = 1)	Single fiber activity (*j* = 2)	Strongly decreased recruitment pattern (*j* = 3)	Mildly decreased recruitment pattern (*j* = 4)
1	4	3	0	0	27	7	0	0	0
2	0	7	0	0	28	0	6	1	0
3	0	0	7	0	29	0	3	3	1
4	0	7	0	0	30	7	0	0	0
5	0	1	1	5	31	0	7	0	0
6	0	0	0	7	32	7	0	0	0
7	0	0	2	5	33	0	5	2	0
8	0	5	2	0	34	7	0	0	0
9	0	1	6	0	35	2	4	1	0
10	0	0	6	1	36	0	5	2	0
11	0	0	2	5	37	5	2	0	0
12	0	0	7	0	38	0	4	2	1
13	0	7	0	0	39	3	4	0	0
14	7	0	0	0	40	0	0	0	7
15	3	4	0	0	41	0	0	5	2
16	0	0	2	5	42	0	1	6	0
17	3	4	0	0	43	0	0	7	0
18	1	3	3	0	44	7	0	0	0
19	0	0	0	7	45	0	5	2	0
20	2	0	0	5	46	0	5	2	0
21	0	3	4	0	47	0	0	7	0
22	0	7	0	0	48	7	0	0	0
23	7	0	0	0	49	4	3	0	0
24	0	7	0	0	50	2	5	0	0
25	0	1	6	0	51	7	0	0	0
26	0	0	6	1	52	0	3	4	0
Total (*r*_*ij*_)	27	60	54	41	Total (*r*_*ij*_)	65	62	44	11

We also measured the extent of agreement beyond chance in assignment to category ***j*** proposed by Fleiss [30]. In all rating categories, the seven examiners also achieved a good agreement. The Fleiss’ Kappa values range from 0.52 to 0.74. In the rating category “no activity/electric silence”, almost an excellent agreement among the seven examiners has been achieved. These values are detailed in Table 5.

**Table 5:** Fleiss’ Kappa values for each rating category

Rating category	Fleiss’ Kappa for individual categories
No activity/electric silence	0.74
Single fiber activity	0.52
Strongly decreased recruitment pattern	0.54
Mildly decreased recruitment pattern	0.69

When analyzing the differences between “strongly decreased recruitment pattern”, “mildly decreased recruitment pattern”, and “normal/dense recruitment pattern”, the inter-rater reliability was much worse.

## Discussion

VFP accounts for an important part of clinical workload in an ENT department. In a university department in Germany, Austria and Switzerland VFP was diagnosed eight times per month, which emphasizes the magnitude of the problem in clinical routine [10]. Wu and Sulica also reported the exact same prevalence for US American laryngology experts [9]. In daily clinical examination of voice disorders, laryngoscopic or videostroboscopic examination is still the most frequently used diagnostic method, although LEMG is recognized as a valuable diagnostic tool for more than 60 years, especially in differentiating neurogenic from structural causes for vocal fold immobility. Although LEMG is the best tool for diagnosing laryngeal paresis objectively and possesses a high predictive value for the outcome of VFP with poor prognosis, many laryngologists still do not routinely used it. The causes might be lacking of agreement on methodology, interpretation, validity and clinical application of LEMG [10].

In this study, we could achieve a reasonable inter-rater reliability among rater pairs and all of the seven raters in general, despite that using Cohen’s Kappa 23.80% and 14.29% of rater pairs achieved only fair agreement in the category “single fiber activity” and “strongly decreased recruitment pattern”, respectively. Whereas assessing the inter-rater reliability between the 7 examiners using Fleiss’ Kappa, it showed good agreement among the raters. In the rating category “no activity/electric silence”, the best agreement among the raters could be observed, while in the rating category “single fiber activity” the least agreement among the observers. This might be attributed to the more difficult differentiation of the latter signal pattern or a not precise enough definition. Also the training level of the raters could explain the differences in the interpretation. Though the inter-rater agreement was acceptable, this indicates that, the way on how to interpret the LEMG is still imperfect. Till now, most classifications describing spontaneous and voluntary EMG characteristics are of descriptive or semi-quantitative nature [17]. Automated signal pattern recognition by validated software algorithms are requested, but not yet well established for LEMG [36]. Thus, the interpretation of the LEMG findings is still considered to be subjective and might account for the different interpretation of the LEMG findings among the raters [37].

LEMG is a valuable diagnostic tool for investigation the causes of vocal fold immobility and estimating the degree of laryngeal nerve damage in laryngeal paralysis. The laryngologist can use this information to make more rational decisions regarding the type and timing of phonosurgery in patients with laryngeal paralysis [37]. Even further, patient counseling on novel therapy options can be performed more sophisticated, if the prognosis on nerve regeneration and restoring of vocal fold function can be estimated by the examiner/physician [38].

For example, the detection of laryngeal synkinesis is of importance for alternative therapy concepts like botulinum toxin infection, electric laryngeal stimulation or laryngeal pacing [39]. Botulinum toxin might weaken the M. thyroarytaenoideus in episodic dyspnoea attacks. Electrical stimulation may promote the specificity of reinnervation of denervated laryngeal muscles. This is an important finding, since 70% of patients having bilateral VFP become synkinetically paralyzed despite successful reinnervation [40].

LEMG is also the only method to show if a subtle vocal fold motion asymmetry is due to a neurologic insult and affords information regarding the sidedness of the abnormality, which may not be obvious from laryngoscopic examination [41].

In summary, a good inter-rater agreement between the participating neurolaryngologists was achieved in the interpretation of LEMGs. For further improvement, the provision of refined definitions of the LEMG rating categories is recommended. We believe that precise interpretation of LEMG signals provides key understanding of the spectrum of the neurogenic causes of vocal fold movement impairment and in consequence plays a major part in the decision on current and emerging therapeutic approaches. Expert agreement on signal interpretation is also needed to establish a solid basis for the development of software-based pattern recognition algorithms that might simplify and, therefore, encourage and spread the clinical application of this valuable diagnostic tool. However, more opportunities of training and workshops should be offered and work on objective quantification methods should be encouraged. A comprehensive network of applied clinical LEMG diagnostic routine should be strived for in ENT and Phoniatric Departments to provide the best basis for therapeutic decisions.

## Conclusion

Overall, LEMG is a useful diagnostic tool for advanced diagnostic evaluation of laryngeal pareses and can play a key role in identifying the optimum therapeutic spectrum for each individual patient. Due to the preceding training of the raters, an acceptable inter-rater agreement could be achieved. Yet to further improve inter-rater agreement among neurolaryngologists and encourage a broader use of LEMG as a diagnostic tool, more instructional and hands-on courses should be offered. Also, as LEMG interpretation is still semi-quantitative and subjective, more experience is needed to achieve a better inter-rater agreement to refine the method, establish clearer definitions of the rating categories and provide diagnostic criteria for future diagnostic algorithms for computer-assisted examinations. In the near future, the LEMG should become one of the gold-standard diagnostic tools for examination of vocal fold movement disorders.
